# Supplementary material for: A hydrogen sulphide-responsive and depleting nanoplatform for cancer photodynamic therapy
Source: Nat Commun. 2022 Mar 30;13:1685. doi: 10.1038/s41467-022-29284-7 (PMC8967875; doi:10.1038/s41467-022-29284-7)
Supplement: Supplementary file 1 — Supplementary Information [file 41467_2022_29284_MOESM1_ESM.pdf]

Supplementary Information

A Hydrogen Sulphide-responsive and Depleting Nanoplatform for Cancer Photodynamic Therapy

Yuqi Zhang[1], Jing Fang[1], Shuyue Ye[1], Yan Zhao[1], Anna Wang [1], Qiulian Mao[1], Chaoxiang Cui[1], Yali Feng[1], Jiachen Li[1], Sunao Li[2], Mingyang Zhang[2], Haibin Shi[1*]

[1]State Key Laboratory of Radiation Medicine and Protection, School for Radiological and Interdisciplinary Sciences (RAD-X) and Collaborative Innovation Center of Radiation Medicine of Jiangsu Higher Education Institutions, Soochow University, Suzhou, Jiangsu, China.
[2]Department of Forensic Sciences, School of Basic Medicine and Biological Sciences, Soochow University, Suzhou, China.

*e-mail: hbshi@suda.edu.cn

Content

Supplementary Figure. 1. 3
Supplementary Figure. 2.……………………3
Supplementary Figure. 3. 4
Supplementary Figure. 4. 5
Supplementary Figure. 5. 5
Supplementary Figure. 6. 5
Supplementary Figure. 7. 6
Supplementary Figure. 8. 6
Supplementary Figure. 10. 8
Supplementary Figure. 11....................8
Supplementary Figure. 12. 9
Supplementary Figure. 13. 10
Supplementary Figure. 14. 10
Supplementary Figure. 15. 11
Supplementary Figure. 16. 11
Supplementary Figure. 17. 12
Supplementary Figure. 18. 12
Supplementary Figure. 19. 12
Supplementary Figure. 20. 13
Supplementary Figure. 21. 13
Supplementary Figure. 22. 13
Supplementary Figure. 23. 14
Supplementary Figure. 24. 14
Supplementary Figure. 25. 15
Supplementary Figure. 27. 16
Supplementary Figure. 28. 17
Supplementary Figure. 29. 17
Supplementary Figure. 30. 18
Supplementary Figure. 31. 18

Supplementary Figure. 32. 19
Supplementary Figure. 33. 19
Supplementary Figure. 34. 20
Supplementary Figure. 35. 20
Supplementary Figure. 36. 21
Supplementary Figure. 37. 21
Supplementary Figure. 38. 22

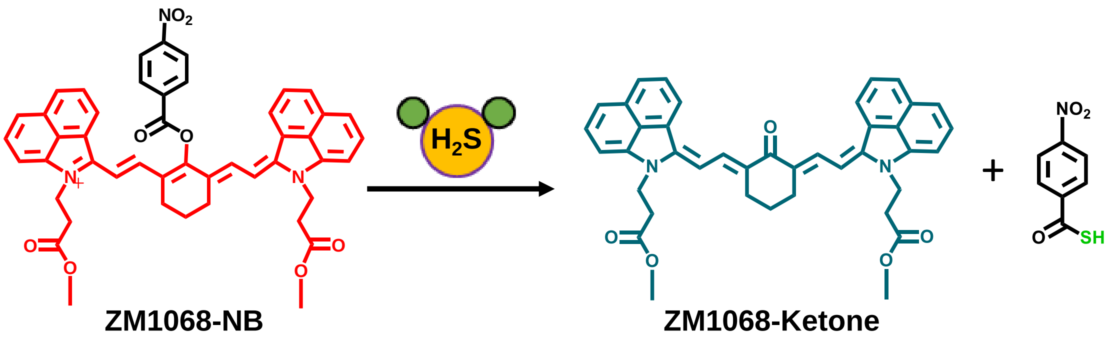

Supplementary Figure. 1. The reaction mechanism of ZM1068-NB to H_2S.

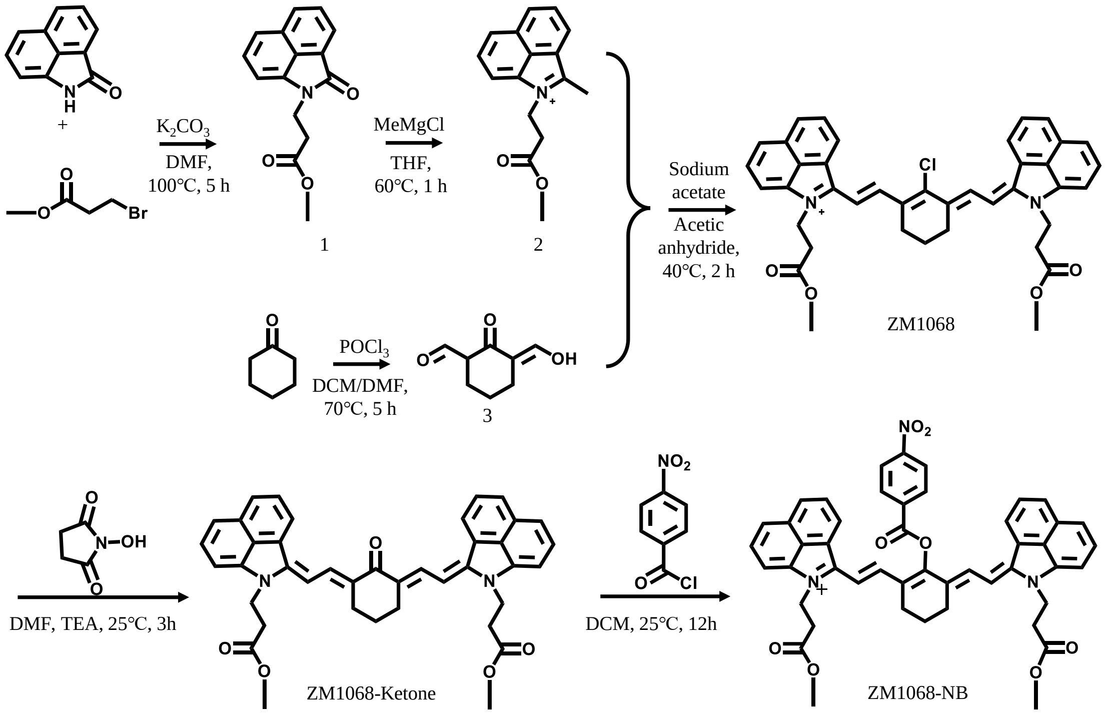

Supplementary Figure. 2. Synthetic scheme of NIR-II dye ZM1068-NB.

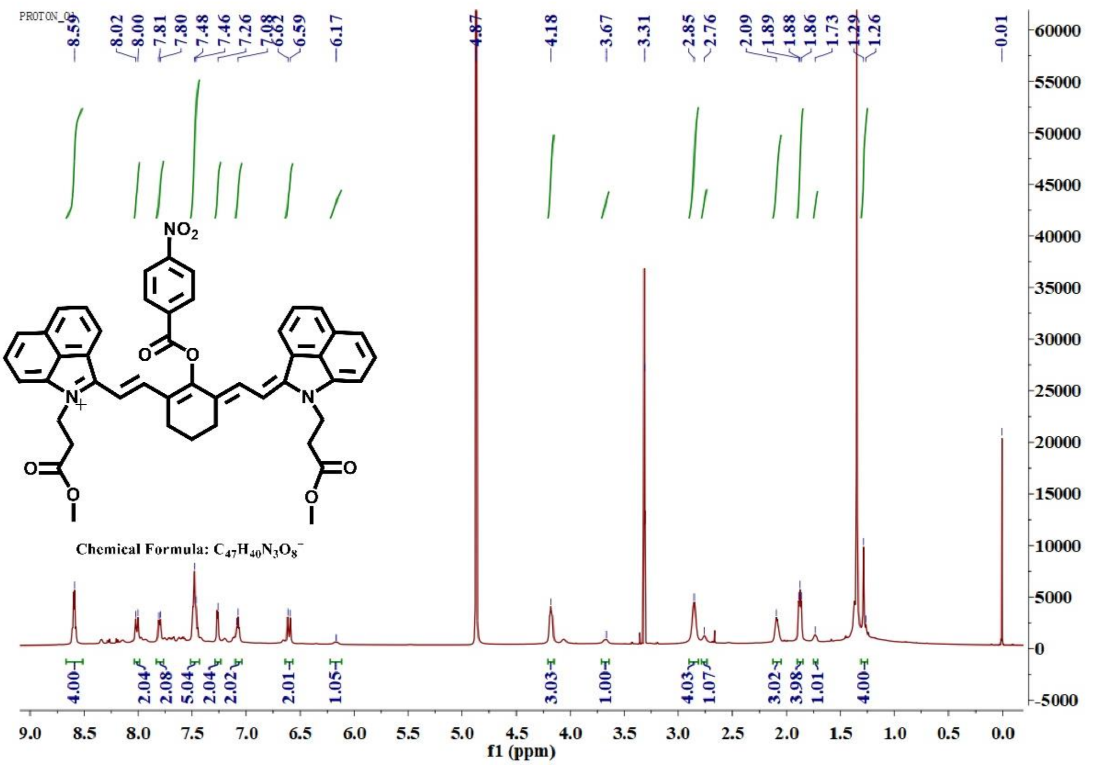

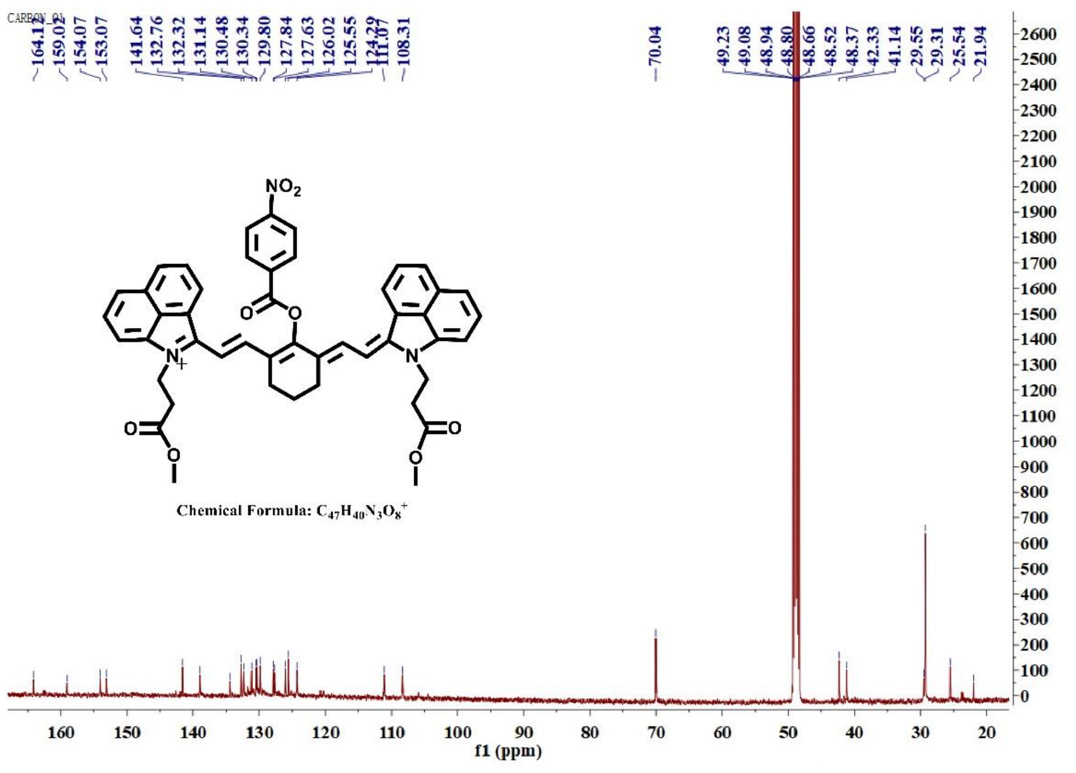

Supplementary Figure. 3. ^{1}H and ^{13}C-NMR spectrometry of ZM1068-NB.

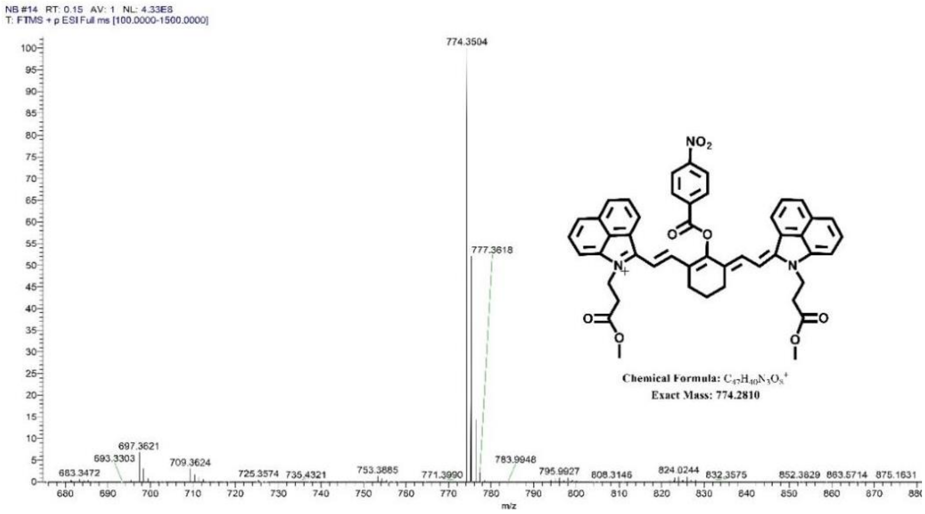

Supplementary Figure. 4. MS spectrum of ZM1068-NB.

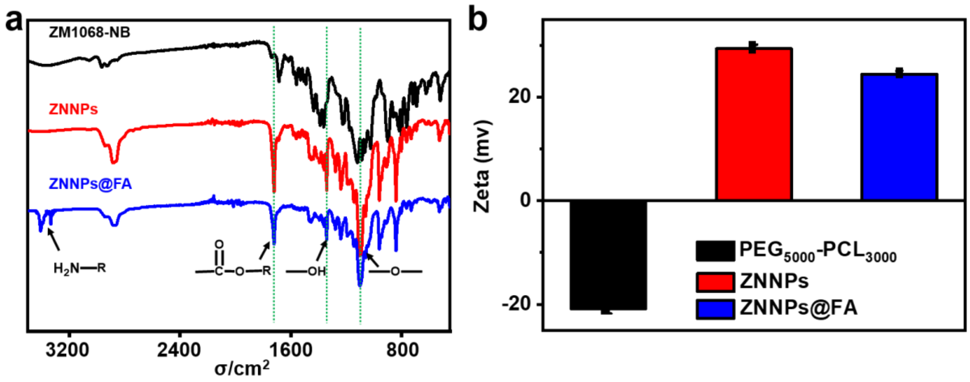

Supplementary Figure. 5. FTIR spectra and Zeta potential of ZNNPs@FA. **a.** IR profile of ZM1068-NB, ZNNPs, and ZNNPs@FA. **b,** Zeta potential of PEG5000-PCL3000, ZNNPs, and ZNNPs@FA. Data are presented as mean ± s.d. (n = 3). Source data are provided as a Source Data file.

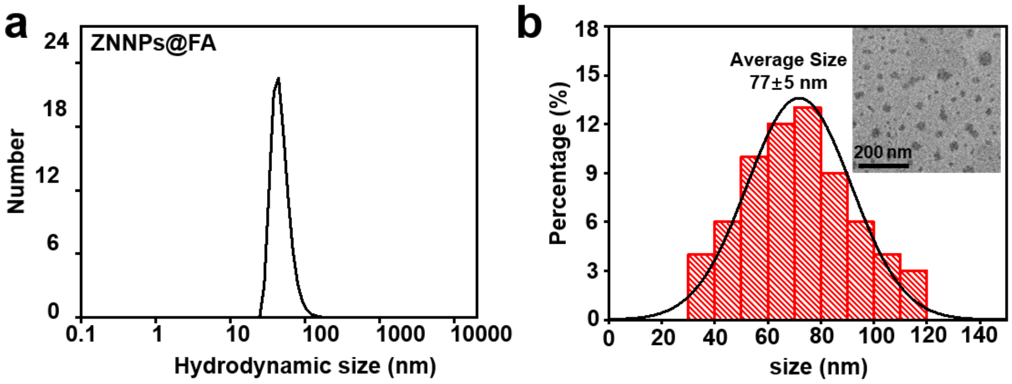

Supplementary Figure. 6. Characterization of particle size for ZNNPs@FA. a, DLS of ZNNPs@FA nanoparticles in PBS buffer (pH = 7.4). **b,** TEM images and average diameter of ZNNPs@FA. Data are presented as mean (n= 3). Source data are provided as a Source Data file.

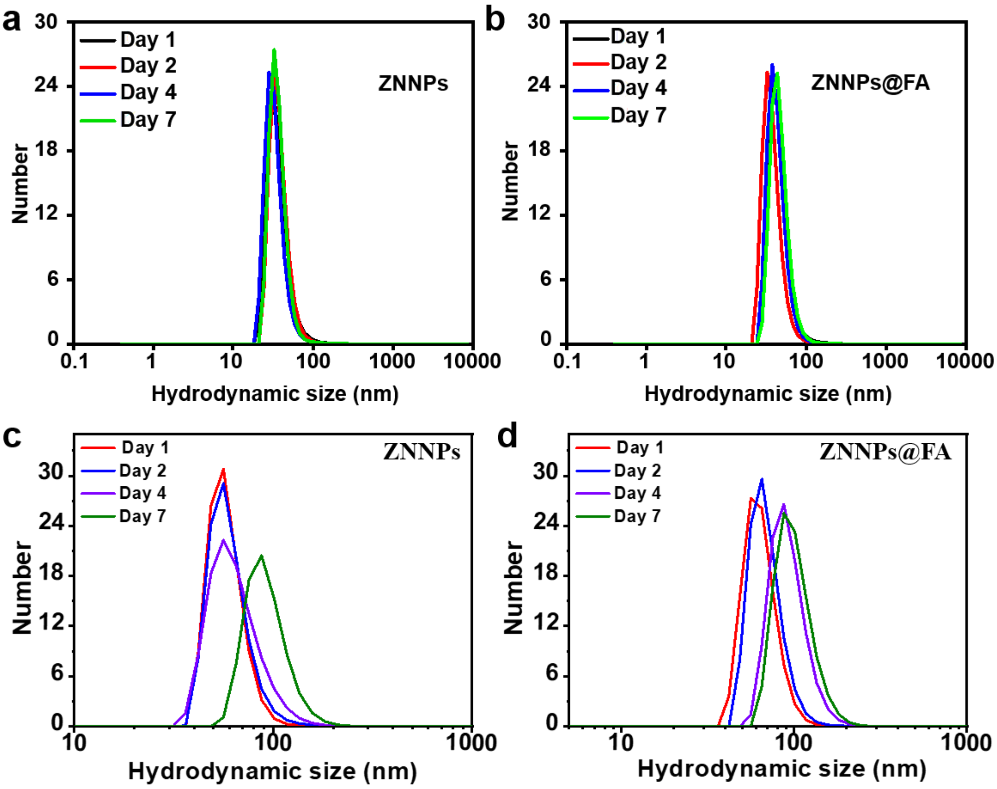

Supplementary Figure. 7. Particle size stability of ZNNPs and ZNNPs@FA. Stability of **a,** ZNNPs and **b,** ZNNPs@FA in PBS buffer (pH =7.4) over 7 days. The stability study of **c,** ZNNPs and **d,** ZNNPs@FA in PBS containing 10% serum at 37°C over one week. Data are presented as mean (n= 3). Source data are provided as a Source Data file.

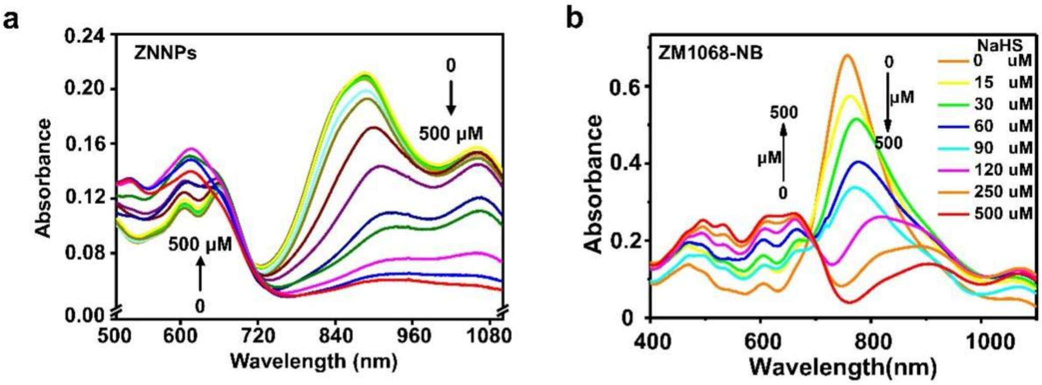

Supplementary Figure. 8. UV absorption changes of ZNNPs and ZM1068-NB under different concentrations of NaHS. Normalized absorption of **a,** ZNNPs (14 μg/mL) and **b,** ZM1068-NB incubated with different concentration NaHS (0 - 500 μM) at room temperature for 10 min. Data are presented as mean (n= 3). Source data are provided as a Source Data file.

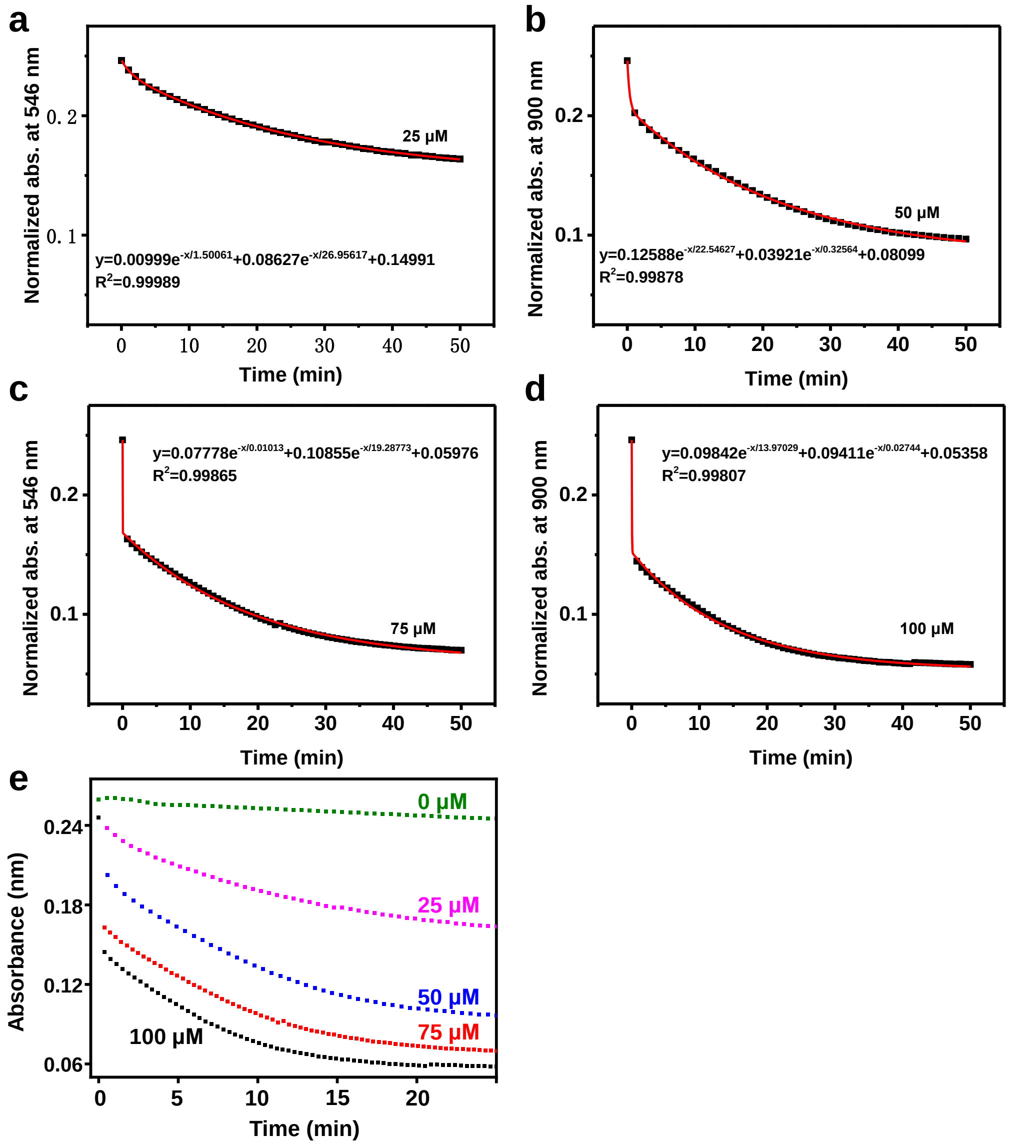

Supplementary Figure. 9. The reaction rate evaluation between ZNNPs and H_2S. Normalized absorption intensity (900 nm) of ZNNPs (14 μg/mL) following incubation with various concentrations of NaHS in PBS buffer (pH 7.4) at 25°C. The concentration of NaHS is **a**, 25 μM, **b**, 50 μM, **c**, 75 μM, **d**, 100 μM. **e**, The combined collection of a-d. Data are presented as mean (n= 3). Source data are provided as a Source Data file.

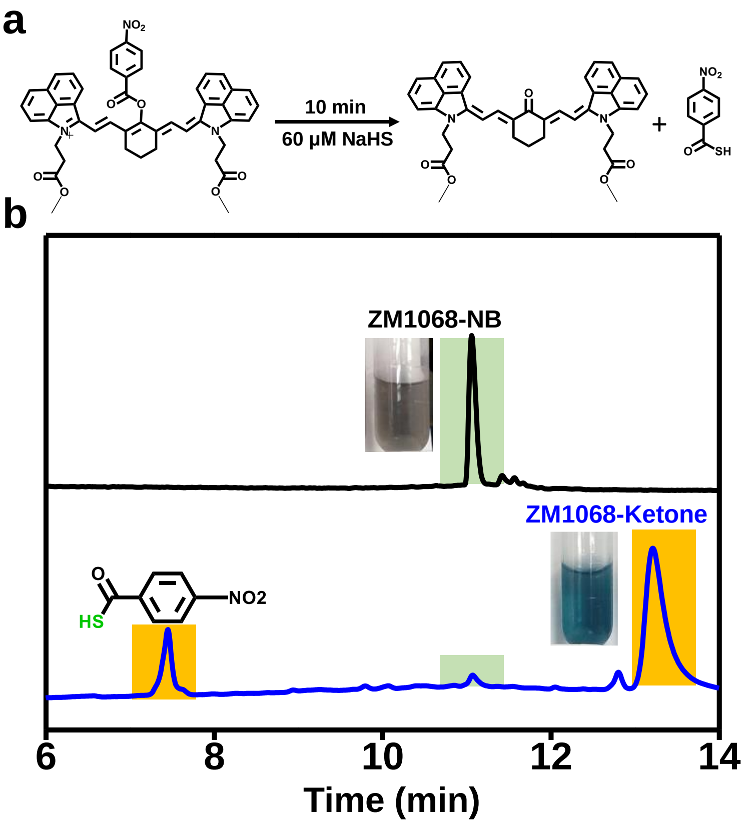

Supplementary Figure. 10. HPLC monitoring of ZNNPs and H_2S reaction. a, The reaction mechanism of ZM1068-NB to H_2S. **b,** Verification of the nucleophilic substitution reaction between ZM1068-NB and H_2S using HPLC. Source data are provided as a Source Data file.

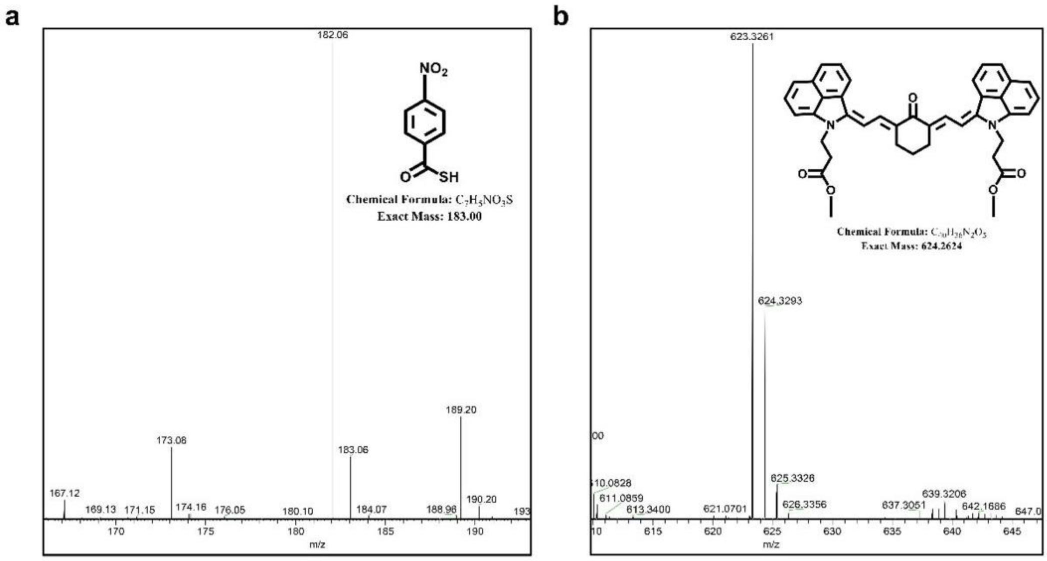

Supplementary Figure. 11. Monitoring of the reaction between ZM1068-NB and H_2S by Q Exactive™ MS measurements. a, Mass spectrometry of reaction products p-Nitrothiobenzoic S-acid. **b,** Mass spectrometry of reaction products ZM-1068-Ketone.

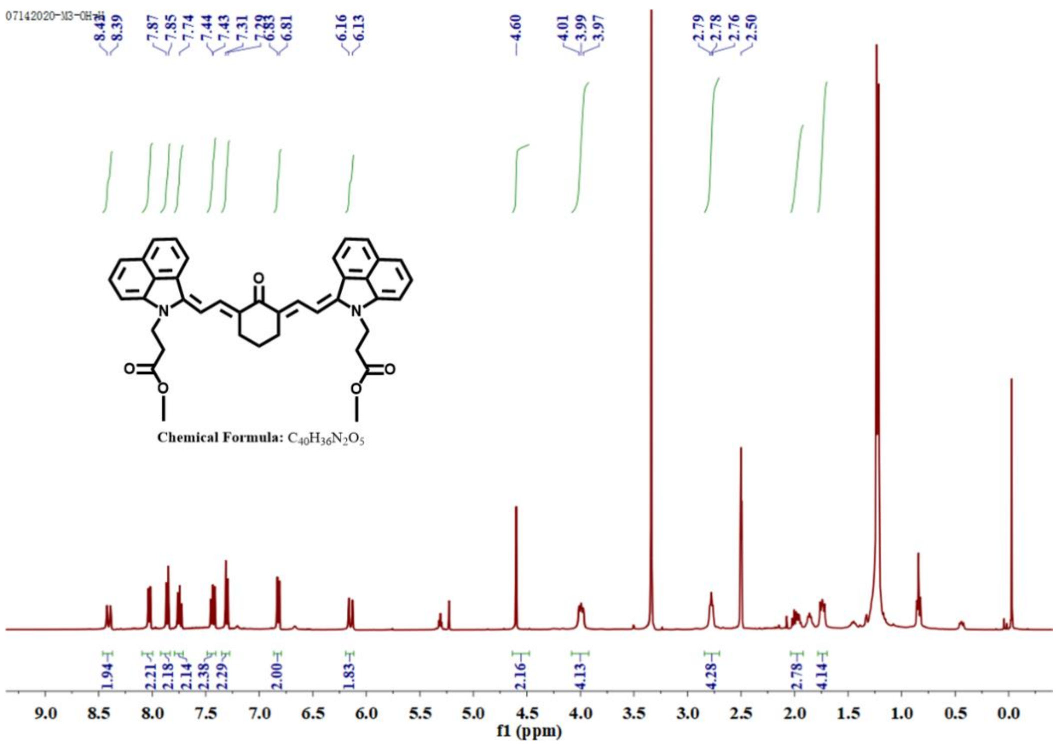

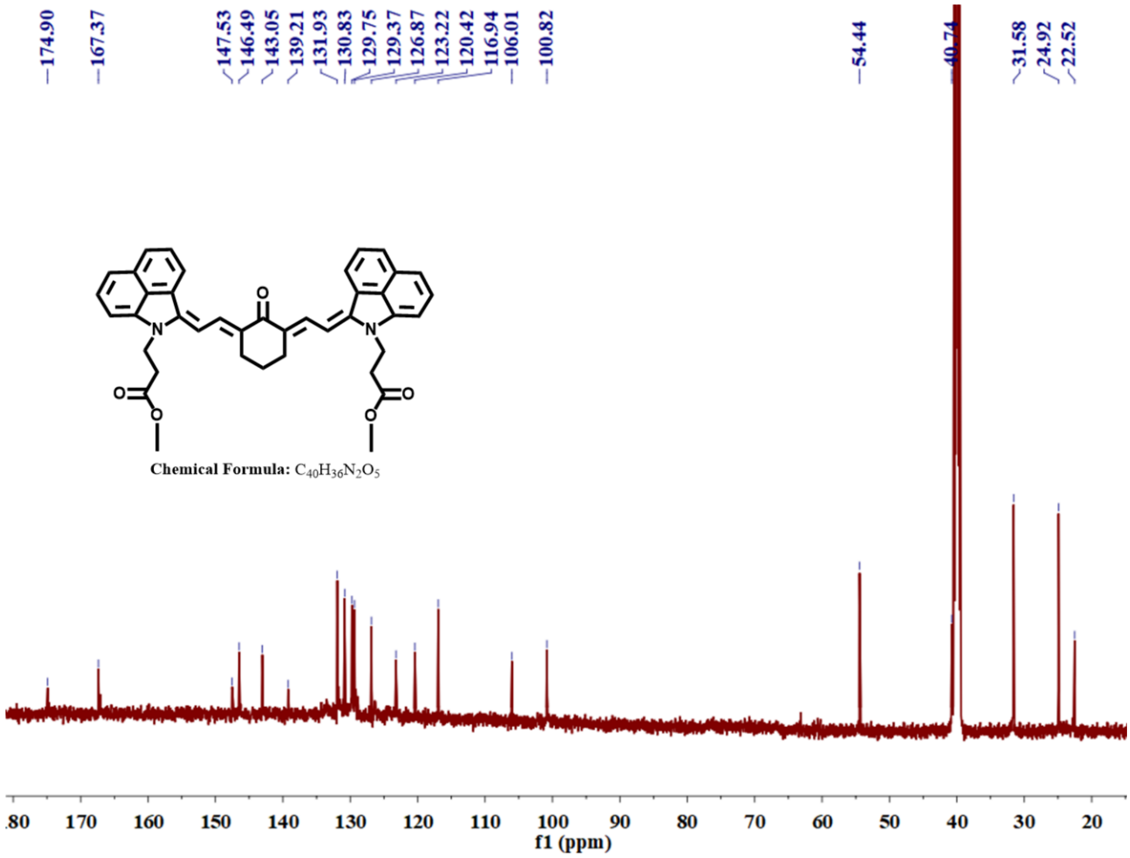

Supplementary Figure. 12. ^{1}H and ^{13}C NMR spectra of compound ZM1068-ketone.

a

	QY(Φ) *Ex*=660nm	QY(Φ) *Ex*=808nm
ZNNPs	1.1%	0.1%
ZNNPs+NaHS (500 μM)	17.2%	0.006%

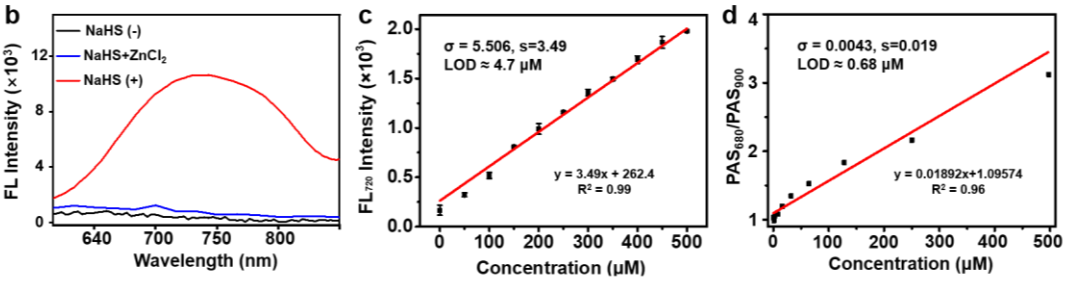

Supplementary Figure. 13. Change of fluorescence quantum yield of ZNNPs before and after reaction with H_2S and the detection limit of ZNNPs to H_2S. a, Fluorescence quantum yields of ZNNPs and ZNNPs with NaHS (500 μM) were determined in PBS (pH 7.4). NIR II dye IR-26 (Φ = 0.05%) and Cy5.5 (Φ = 23%) was used as the reference. All the measurements were carried out at room temperature. **b,** Fluorescence spectra of ZNNPs (14 μg/mL) upon incubation with NaHS (100 μM) [denoted as NaHS (+)], a mixture of NaHS (100 μM) and its scavenger $ZnCl_2$ (200 μM) [denoted as NaHS+$ZnCl_2$] at 37°C for 10 min in PBS buffer (pH 7.4). Determination of the detection limit of ZNNPs toward H_2S. **c,** The linear relationship between the fluorescence intensity at 720 nm and the various concentrations of NaHS ranging from 0 to 500 μM. **d,** The linear relationship between the ratiometric photoacoustic signals (PAS_{680}/PAS_{900}) and the various concentrations of NaHS ranging from 0 to 500 μM. Data are presented as mean ± s.d. (n = 3 independent samples). Source data are provided as a Source Data file.

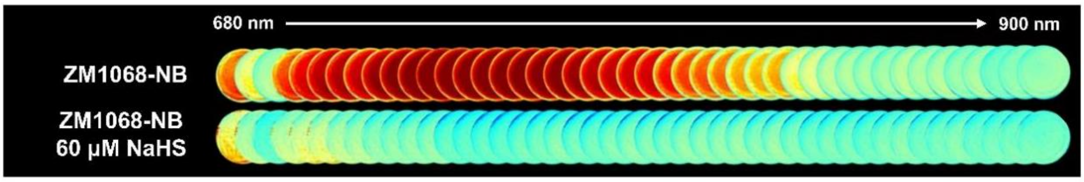

Supplementary Figure. 14. The photoacoustic images (680 nm - 900 nm) of ZM1068-NB (14 μg/mL) incubated with NaHS (0 and 60 μM) at room temperature for 10 min. Data are presented as images (n= 1).

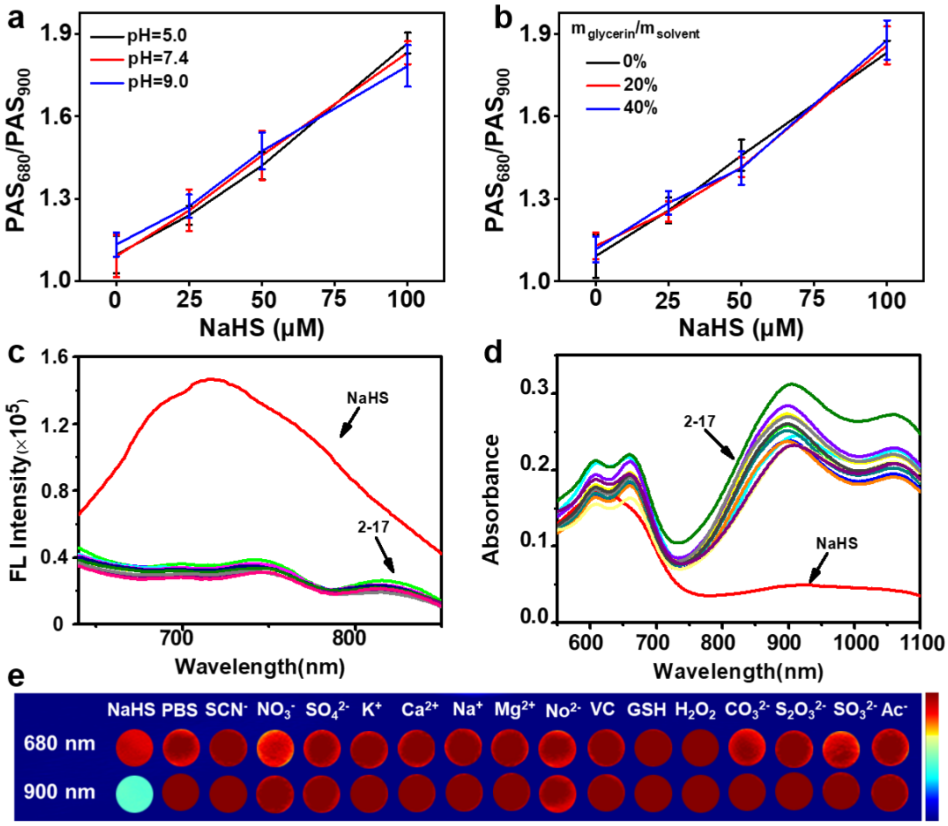

Supplementary Figure. 15. Specific detection of ZNNPs to H_2S. The effect of pH **a** and viscosity **b** on the ratiometric photoacoustic signal (PA_{680}/PA_{900}) responsiveness of ZNNPs (14 μg/mL) toward NaHS in PBS buffer. **c,** Fluorescence spectra, **d,** normalized absorption and **e,** photoacoustic image. Data are presented as mean ± s.d. (n = 3). Source data are provided as a Source Data file.

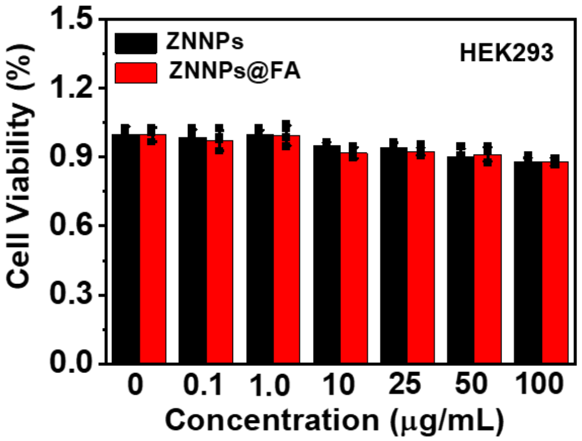

Supplementary Figure. 16. Viability of HEK293 cells after 24 h of treatment with different concentrations of ZNNPs or ZNNPs@FA. Data are presented as mean ± s.d. (n= 4 independent cell pellets). Source data are provided as a Source Data file.

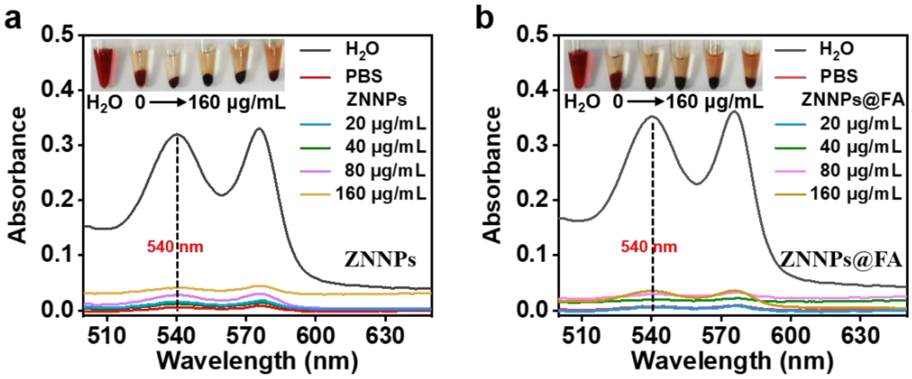

Supplementary Figure. 17. Hemolytic test of nanoprobes. a, ZNNPs and **b,** ZNNPs@FA at different concentrations. Data are presented as mean (n= 3). Source data are provided as a Source Data file.

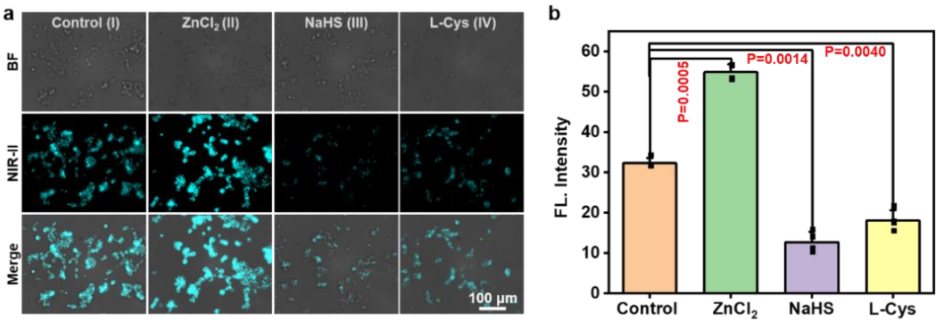

Supplementary Figure. 18. NIR-II imaging of HCT116 cells treated with ZNNPs (20 µg/mL). a, NIR-II confocal fluorescence images and **b**, quantitative fluorescence intensity of HCT116 cells that were treated with PBS buffer, $ZnCl_2$ (40 µg/mL, 10 min), NaHS (100 µM, 1 h), and L-Cys (24 µg/mL, 1 h). Data are presented as mean ± s.d. (n= 4 independent cell pellets). Statistical differences were analyzed by Student's two-sided t-test. Source data are provided as a Source Data file.

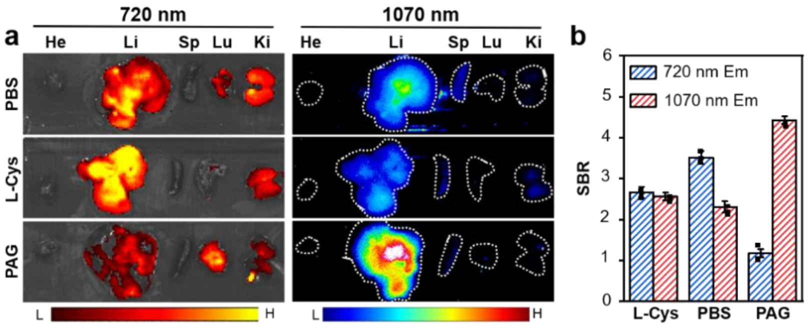

Supplementary Figure. 19. Fluorescent imaging of mouse main organs. Representative images **a,** and average fluorescence intensity **b,** of main organs (He: heart, Li: liver, Sp: spleen, Lu: lung, Ki: kidneys) resected from mice 90 min post injection (i.v.) of ZNNPs at 720 and 1070 nm. PBS (100 µL), L-Cys (6 mM, 100 µL), and PAG (2 mg/mL, 100 µL). Data are presented as mean ± s.d. (n= 3 independent mice). Source data are provided as a Source Data file.

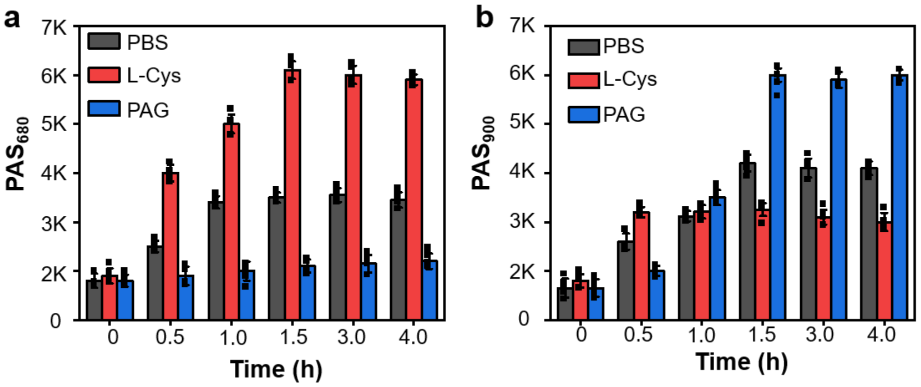

Supplementary Figure. 20. Average photoacoustic signals of mouse main organs. a, 680 nm and **b,** 900 nm in region of liver treated with ZNNPs (10 mg/kg, i.v.). PBS (100 μL), L-Cys (6 mM, 100 μL), and PAG (2 mg/mL, 100 μL). Data are presented as mean ± s.d. (n = 5 independent mice). Source data are provided as a Source Data file.

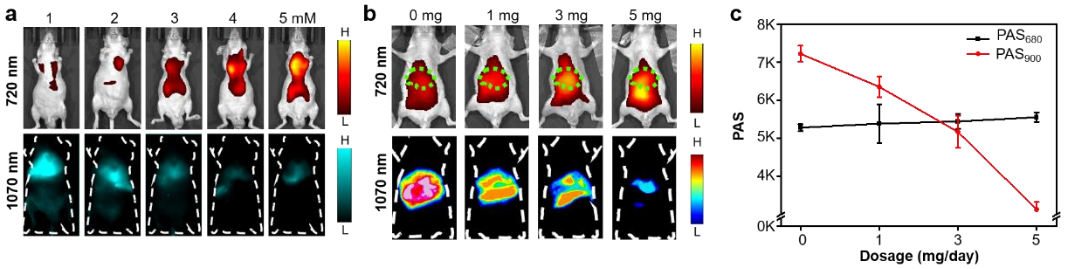

Supplementary Figure. 21. Non-invasive imaging of hepatic H_2S in mouse livers. Fluorescence images at 720 and 1070 nm of various amount of L-Cys (0, 1, 2, 3, 4, 5 mM, 100 μL) **a,** or metformin (0, 1, 3, and 5 mg/day for 7 days) **b,** treated mice following i.v. injection of ZNNPs (10 mg/kg) (n = 5). **c,** Average photoacoustic signal intensity at 680 nm and 900 nm of metformin-treated mouse following i.v. injection of ZNNPs (10 mg/kg). Data are presented as mean ± s.d. (n = 5 independent mice). Source data are provided as a Source Data file.

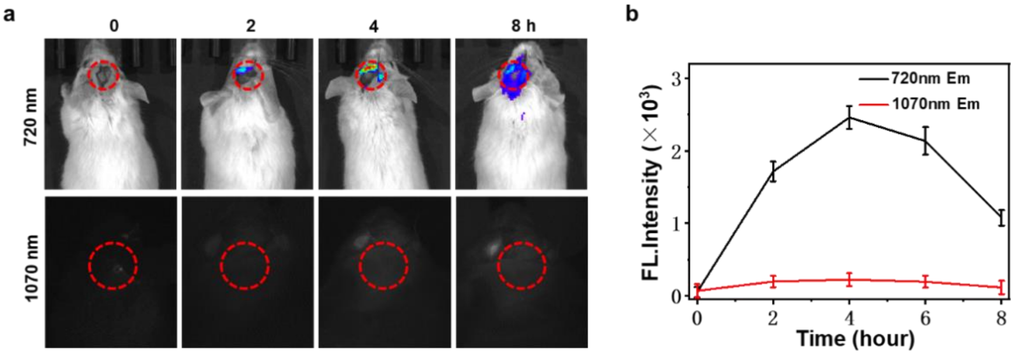

Supplementary Figure. 22. Non-invasive imaging of endogenous H_2S in cerebral hemorrhage mice. a, Fluorescence images at 720 and 1070 nm of ICH mice injected (i.v.) ZNNPs (10 mg/kg) (n = 3). **b,** Quantitative fluorescence intensities of a. Data are presented as mean ± s.d. (n = 4 independent mice). Source data are provided as a Source Data file.

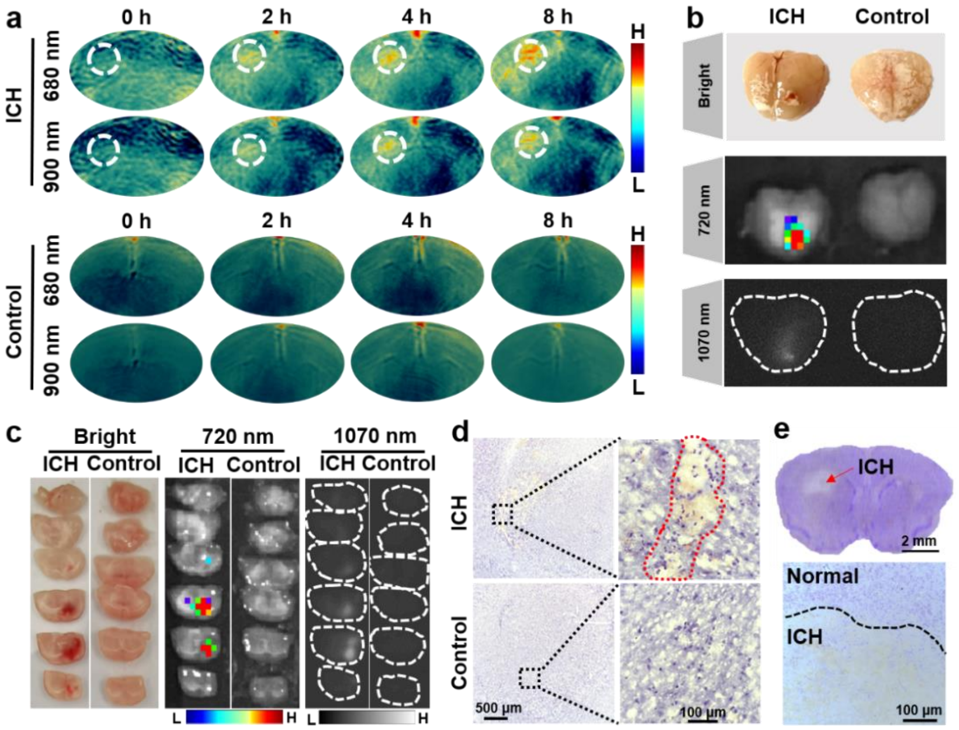

Supplementary Figure. 23. Comparison of the blood-brain barrier (BBB) permeability between intracerebral hemorrhage (ICH) mice and normal mice (Control). a, PA images of ICH mice and normal mice with i.v. injection of ZNNPs (10 mg/kg), respectively. **b** and **c**, Ex vivo NIR-I (Ex=640 nm, Em=720 nm) and NIR-II (Ex=808 nm, Em=1070 nm) fluorescence imaging of the mouse brains. (d) H&E staining images of ICH mice and normal mice. **e**, Nissl staining images of ICH mouse brain, showing injured region by a lack of staining (Group ICH and Control n=1 mouse respectively).

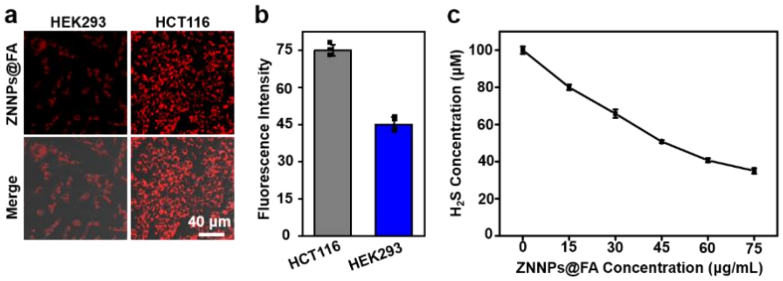

Supplementary Figure. 24. Fluorescence imaging of endogenous H_2S in living cells. a, Fluorescence imaging (Em: 720 nm) of HEK293 and HCT116 cells incubated with ZNNPs@FA (20 μg/mL) for 4 h. **b,** Quantitative fluorescence intensities of (a). **c,** Depletion of intracellular H_2S by ZNNPs@FA treatment. Data are presented as mean ± s.d. (n= 3). Source data are provided as a Source Data file.

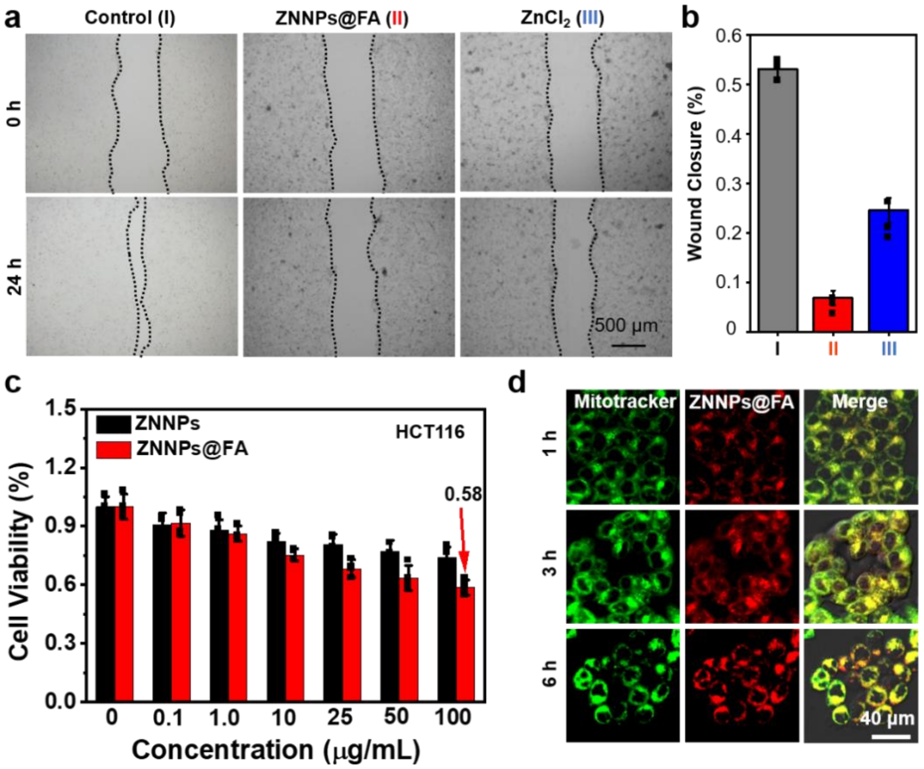

Supplementary Figure. 25. ZNNPs@FA reduces HCT116 cell proliferation via intracellular H_2S depletion. a, Scratch test of HCT116 cells treated with ZNNPs@FA (20 μg/mL) for 4 h, or $ZnCl_2$ (300 μM) for 10 min. **b,** Quantification of scratch test results in (a). **c,** Viability of HCT116 cells treated with different concentrations of ZNNPs or ZNNPs@FA for 24 h incubation. **d,** Co-localization of ZNNPs@FA with mitochondrial tracker. The cells were incubated with ZNNPs@FA (20 μg/mL) for various time points, then washed by PBS for three times followed by incubation with Mito-Tracker Green (100 nM, 1 mL) for 30 min and washed by PBS for fluorescence microscope imaging. Data are presented as mean ± s.d. (n= 4 independent cell pellets). Source data are provided as a Source Data file.

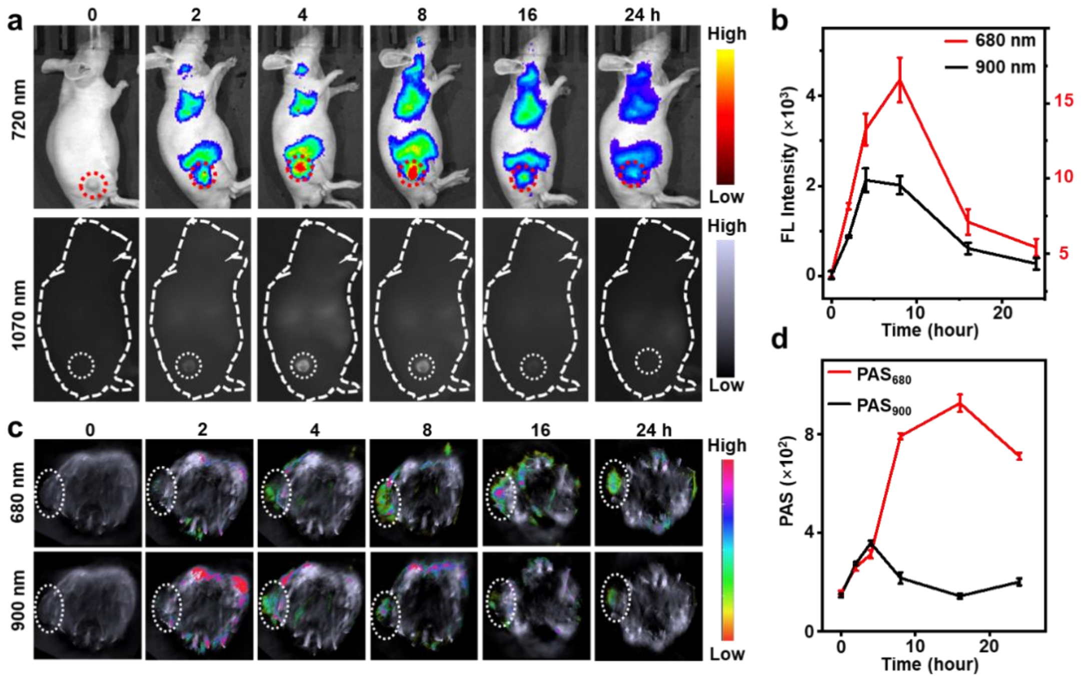

Supplementary Figure. 26. In vivo non-invasive imaging of HCT116 tumors. a, Real-time fluorescent images at 720 and 1070 nm of HCT116 xenograft tumors treated with ZNNPs@FA (100 mg/mL, 200 μL) through tail vail. **b,** The quantification of fluorescent images in (a). **c,** Real-time photoacoustic images at 680 and 900 nm and **d,** quantification of HCT116 xenograft tumor injected with ZNNPs@FA (100 mg/mL, 200 μL) through tail vail. Data are presented as mean ± s.d. (n= 3 independent mice). Source data are provided as a Source Data file.

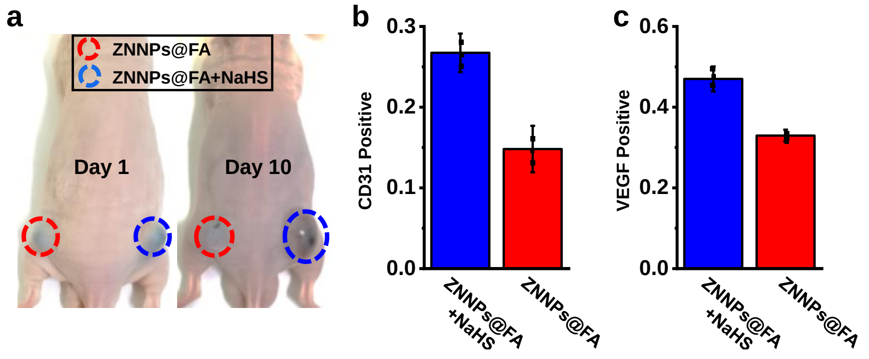

Supplementary Figure. 27. In vivo evaluation of the growth suppression of subcutaneous HCT116 tumors and quantitative fluorescence of immunofluorescence assays. a, The tumors were intratumorally injected with 1.4 mg/mL ZNNPs@FA (20 μL) (left) and 1.4 mg/mL ZNNPs@FA+5 μM NaHS (20 μL) (right) every two days. Data are presented as mean ± s.d. (n= 3 independent mice). Quantitative fluorescence (green) of immunofluorescence staining from antibodies against CD-31 **b**, and VEGF **c** in Figure 6j. Data are presented as mean ± s.d. (n= 3). Source data are provided as a Source Data file.

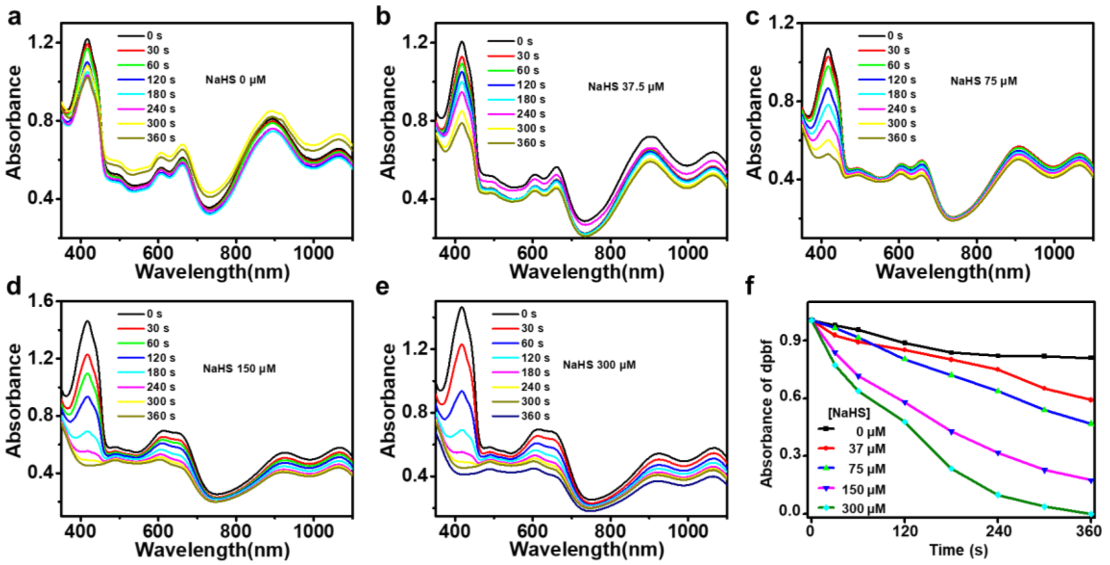

Supplementary Figure. 28. Photodynamic effect of ZNNPs@FA in solution. UV absorption spectra of the mixture **a,** NaHS (0 μM, 2 mL)+ZNNPs@FA (60 μg)+DPBF (43 μg) **b,** NaHS (37.5 μM, 2 mL)+ZNNPs@FA (60 μg)+DPBF (43 μg) **c,** NaHS (75 μM, 2 mL)+ZNNPs@FA (60 μg)+DPBF (43 μg) **d,** NaHS (150 μM, 2 mL)+ZNNPs@FA (60 μg)+DPBF (43 μg) **e,** NaHS (300 μM, 2 mL)+ZNNPs@FA (60 μg)+DPBF (43 μg) over 360 s. **f,** UV absorption changes of DPBF at 415 nm over 360 s. Data are presented as mean (n= 3). Source data are provided as a Source Data file.

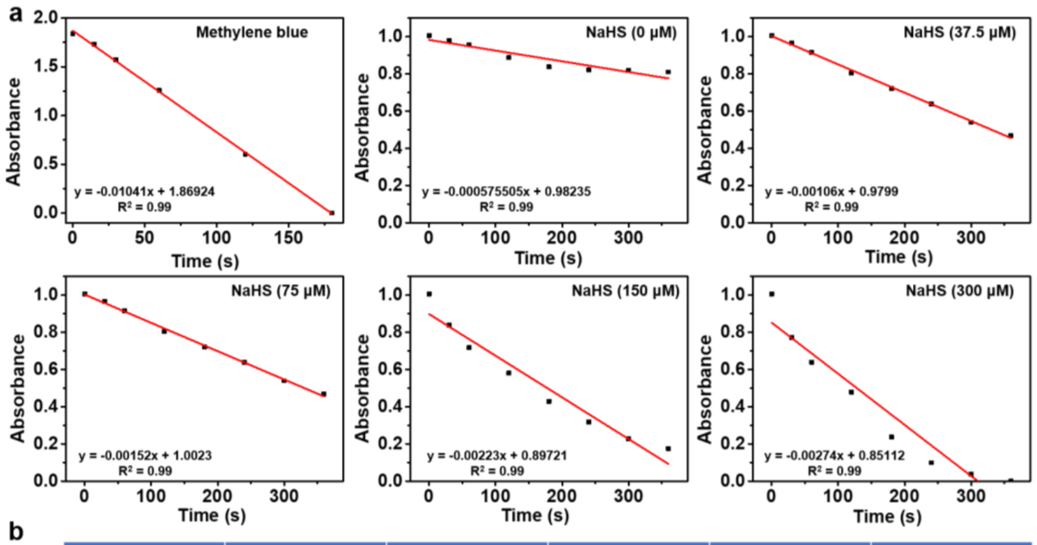

b

NaHS (μM)	0	37.5	75	150	300
$\Phi_{\Delta}(^1O_2)$	1.3%	2.5%	4.0%	4.9%	6.0%

Supplementary Figure. 29. Measurement of singlet oxygen ($^1O_2$) quantum yield for probe ZNNPs@FA. Methylene blue in acetonitrile (Φ_{Δ}= 0.52) was used as the reference. **a,** Curve fitting of DPBF consumption rate in methylene blue (30 μg/mL) solution. **b-f,** Curve fitting of DPBF consumption rate in ZNNPs@FA (30 μg/mL) solution with different concentrations of NaHS (0, 37.5, 75, 150, and 300 μM). (g) $^1O_2$ quantum yields of ZNNPs@FA in the presence of different concentrations of NaHS. Data are presented as mean (n= 3). Source data are provided as a Source Data file.

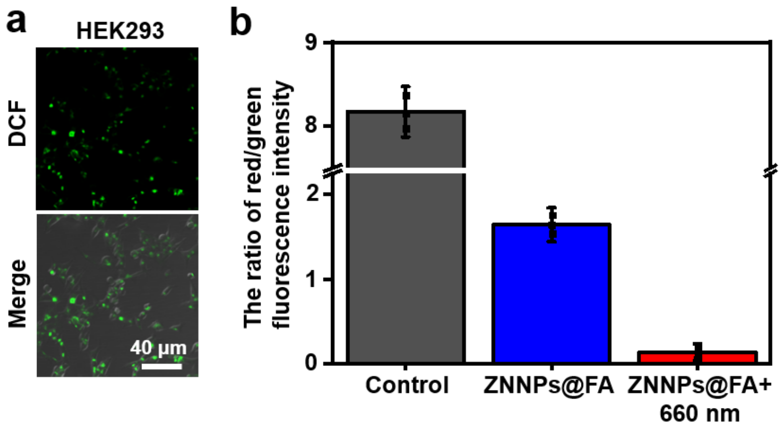

Supplementary Figure. 30. Singlet oxygen production detection in HEK293. a, Confocal images of HEK293 cells that were pretreated with ZNNPs@FA (20 μg/mL) for 4 h and 660 nm laser (50 mW/cm^2) for 3 min followed by DCF-DA treatment. **b,** The ratio of red/green fluorescence intensity in Figure 6c. Data are presented as mean ± s.d. (n= 3 independent cell pellets). Source data are provided as a Source Data file.

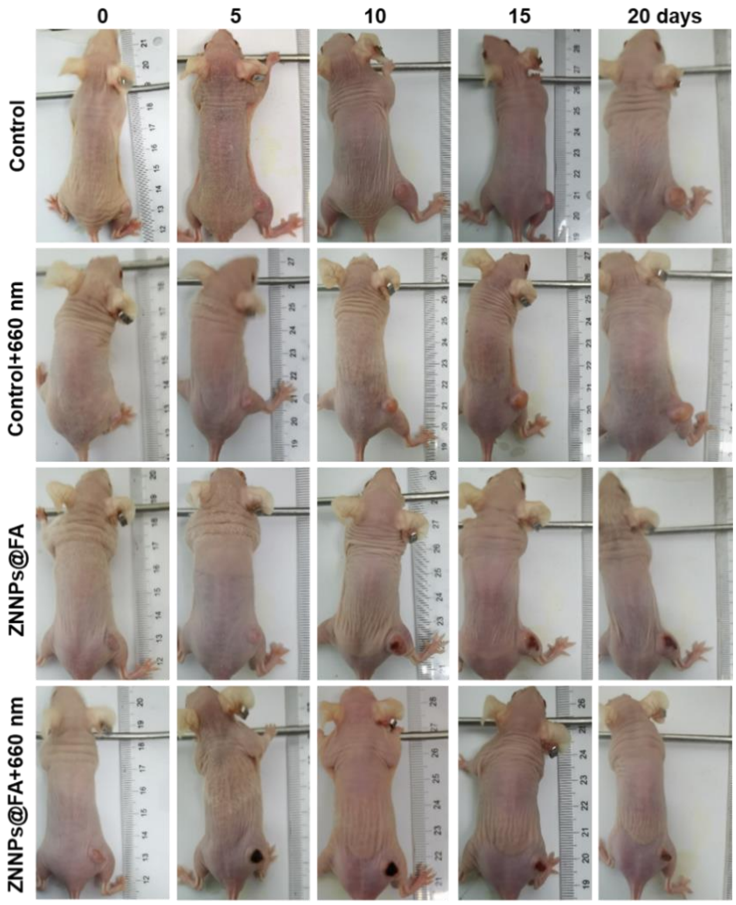

Supplementary Figure. 31. Images of representative mice captured on different days after each treatment of Figure. 8.

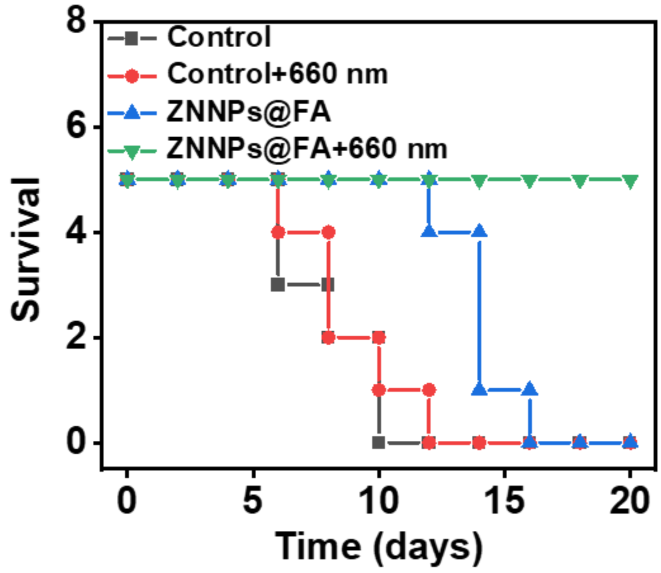

Supplementary Figure. 32. Survival rate of mice for each treatment group of Figure. 8. Data are presented as real number (n= 5 independent mice). Source data are provided as a Source Data file.

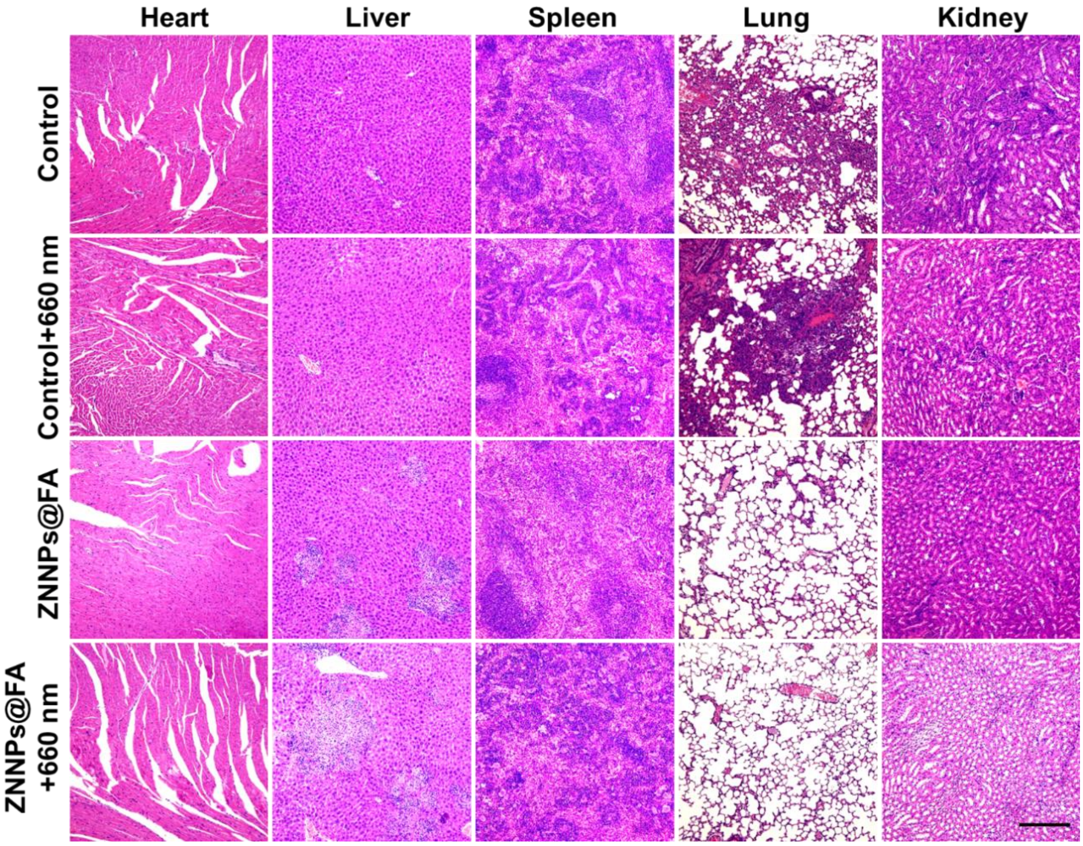

Supplementary Figure. 33. H&E staining of main organs from representative mice captured on 20 days after each treatment of Figure. 8 (n= 5 independent mice). Scale bar: 50 μm.

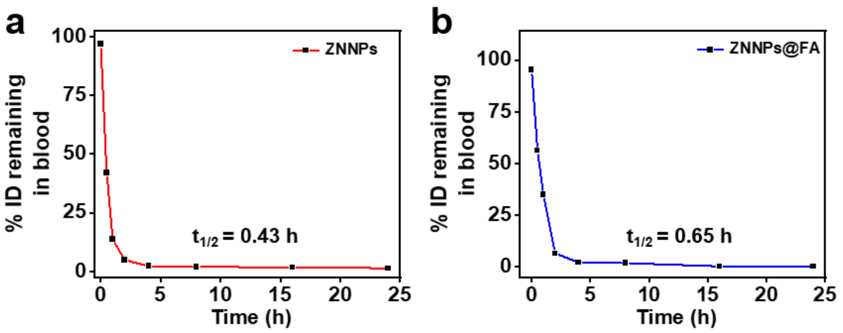

Supplementary Figure. 34. Blood clearance curve. a, ZNNPs ($t_{1/2}$= 0.43 h). **b,** ZNNPs@FA ($t_{1/2}$= 0.65 h). ZNNPs (10 mg/kg) and ZNNPs@FA (10 mg/kg) were i.v. injected into the mice. (100 μL of blood were pretreated by adding 1 μL of 50 mM NaHS solution and incubating at 37℃ for 2 h). Data are presented as mean (n= 3 independent mice). Source data are provided as a Source Data file.

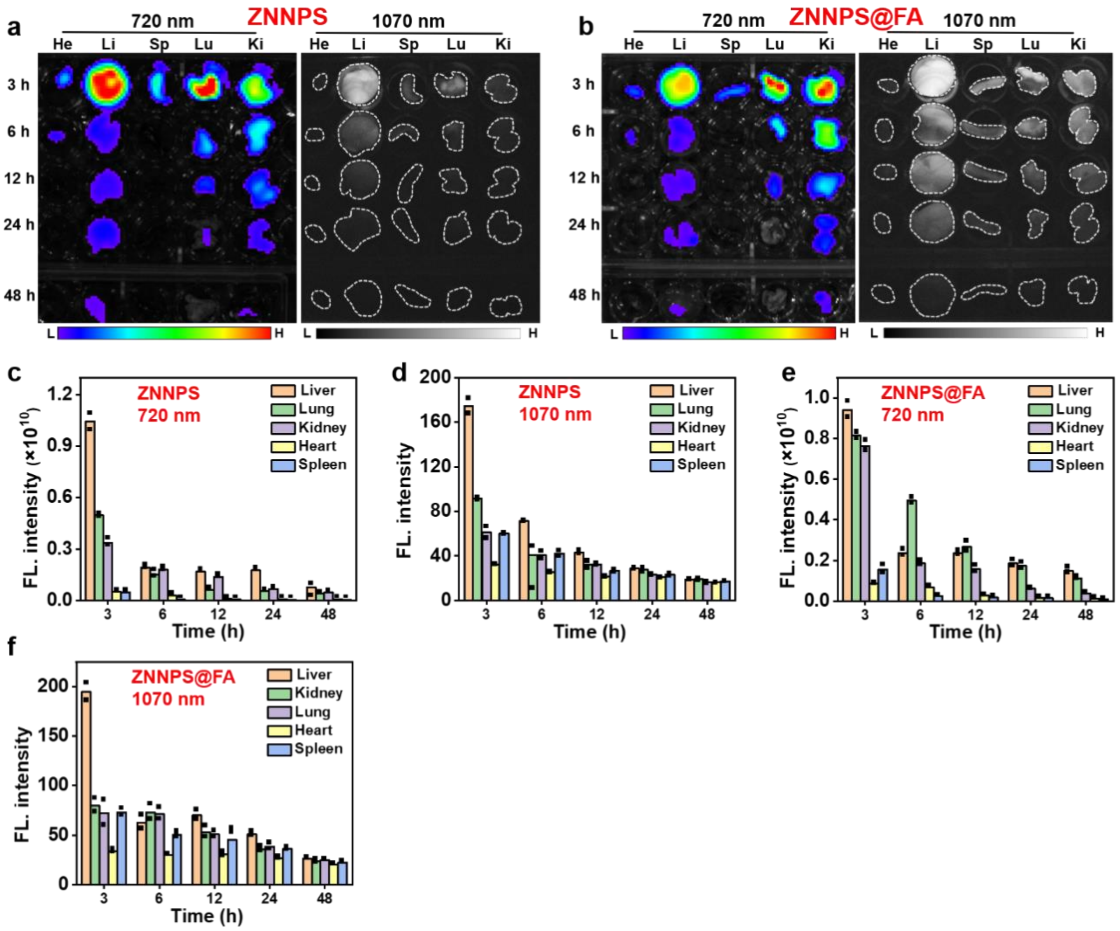

Supplementary Figure. 35. Metabolism of ZNNPs and ZNNPs@FA in different organs. *Ex vivo* real-time NIR-I (E_x/E_m = 640/720 nm) and NIR-II (E_x/E_m = 808/1070 nm) fluorescence imaging of the mice with i.v. injection of **a**, ZNNPs (10 mg/kg) and **b**, ZNNPs@FA (10 mg/kg). **c-f**, The quantification of above fluorescence images in a and b. Data are presented as mean (n= 2 independent mice). Source data are provided as a Source Data file.

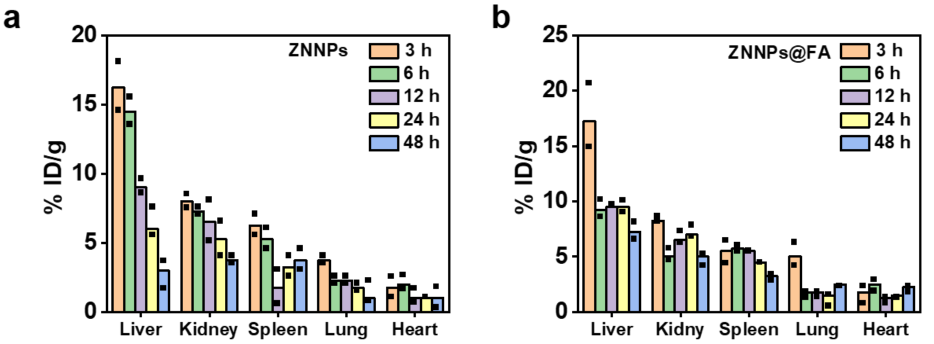

Supplementary Figure. 36. Quantitative analysis of both probes in various organs over time after i.v. injection. a, ZNNPs (10 mg/kg) and **b**, ZNNPs@FA (10 mg/kg) Every 100 μL of organ homogenate was pretreated by adding 1 μL of 50 mM NaHS solution and incubating at 37°C for 2 h. Data are presented as mean (n= 2 independent mice). Source data are provided as a Source Data file.

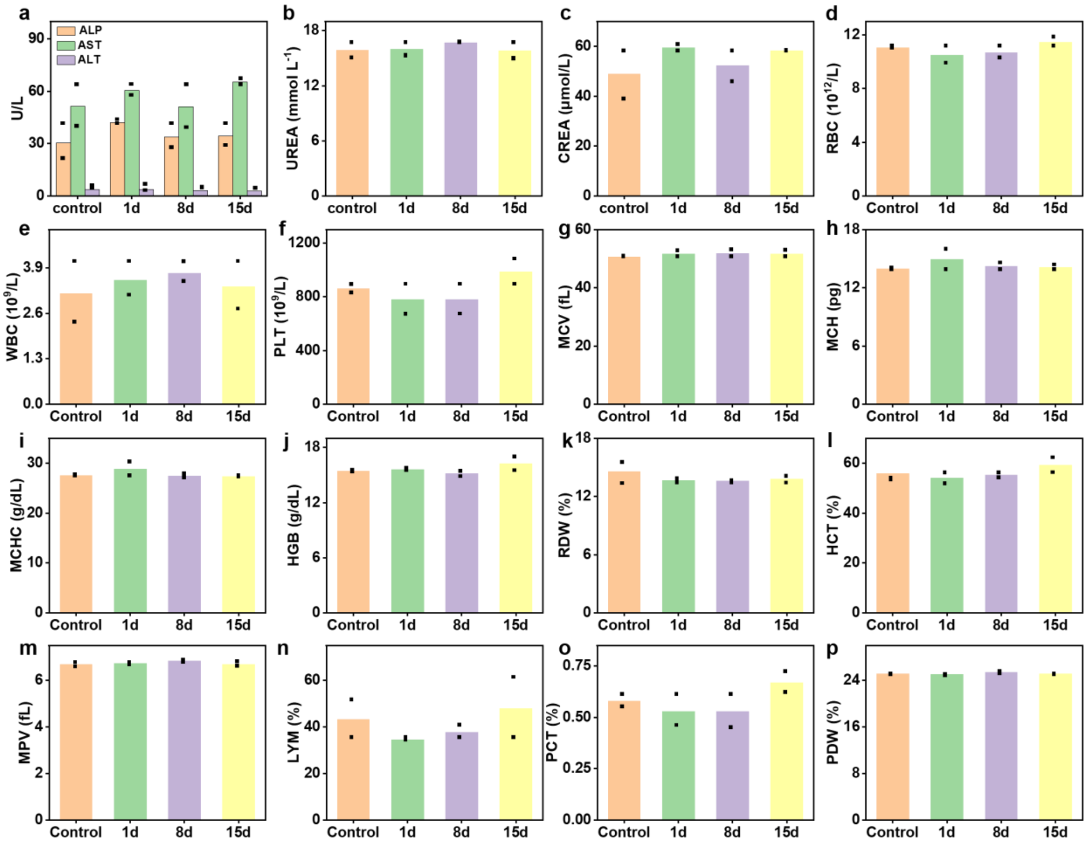

Supplementary Figure. 37. Variations of blood biochemical and blood routine indexes of mice with i.v. injection of ZNNPs (10 mg/kg) at different time points in comparison with non-treated ones (Control). a, Alkaline phosphatase, ALP; aspartate aminotransferase, AST and alanine aminotransferase, ALT. **b**, urea nitrogen, UREA. **c**, creatinine, CREA. **d**, Red blood cells, RBC. **e**, White blood cells, WBC. **f**, Platelets, PLT. **g**, Mean corpuscular volume, MCV. **h**, Mean corpuscular hemoglobin, MCH. **i**, Mean

corpuscular hemoglobin concentration, MCHC. **j**, Hemoglobin, HGB. **k**, Red cell distribution width, RDW. **l**, Hematocrit, HCT. **m**, Mean platelet volume, MPV. **n**, Lymphocyte, LYM. **o**, Plateletcrit, PCT. **p**, Platelet distribution width, PDW. Data are presented as mean (n= 2 independent mice). Source data are provided as a Source Data file.

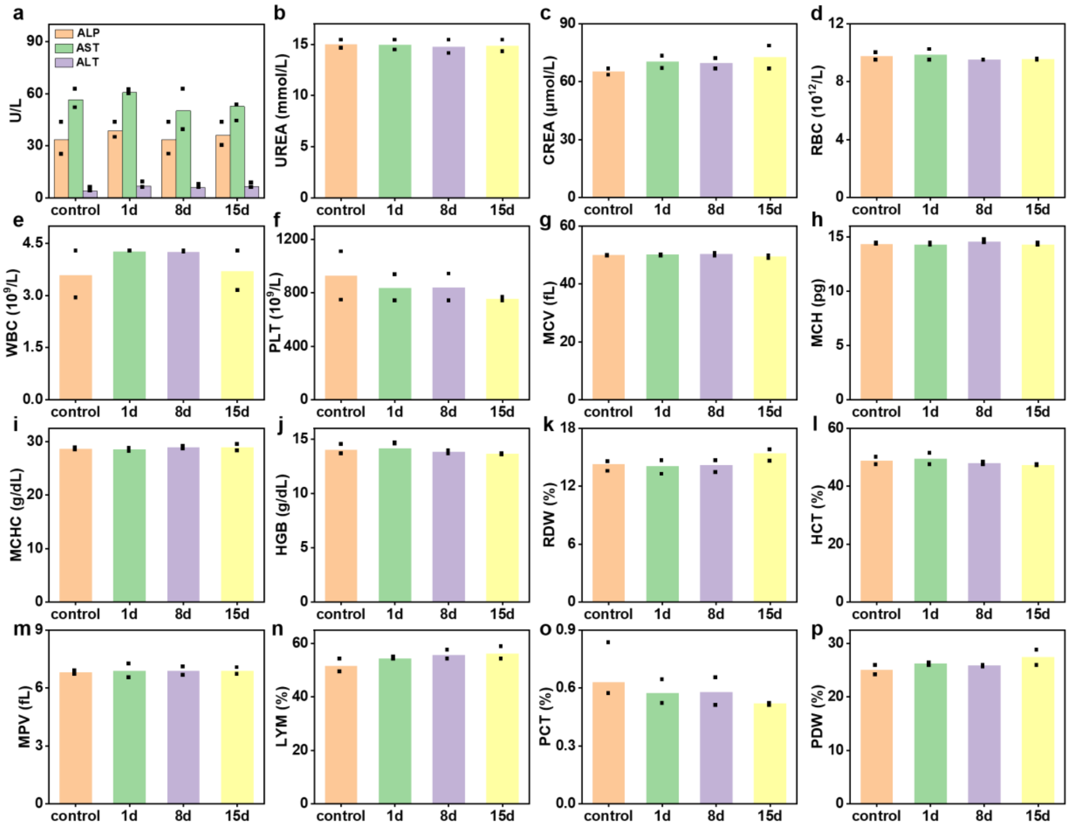

Supplementary Figure. 38. Variations of blood biochemical and blood routine indexes of mice with i.v. injection of ZNNPs@FA (10 mg/kg) at different time points in comparison with non-treated ones (Control). **a**, Alkaline phosphatase, ALP; aspartate aminotransferase, AST and alanine aminotransferase, ALT. **b**, urea nitrogen, UREA. **c**, creatinine, CREA. **d**, Red blood cells, RBC. **e**, White blood cells, WBC. **f**, Platelets, PLT. **g**, Mean corpuscular volume, MCV. **h**, Mean corpuscular hemoglobin, MCH. **i**, Mean corpuscular hemoglobin concentration, MCHC. **j**, Hemoglobin, HGB. **k**, Red cell distribution width, RDW. **l**, Hematocrit, HCT. **m**, Mean platelet volume, MPV. **n**, Lymphocyte, LYM. **o**, Plateletcrit, PCT. **p**, Platelet distribution width, PDW. Data are presented as mean (n= 2 independent mice). Source data are provided as a Source Data file.
