# Supplementary material for: A hydrogen sulphide-responsive and depleting nanoplatform for cancer photodynamic therapy
Source: Nat Commun. 2022 Mar 30;13:1685. doi: 10.1038/s41467-022-29284-7 (PMC8967875; doi:10.1038/s41467-022-29284-7)
Supplement: Supplementary file 2 — Reporting Summary [file 41467_2022_29284_MOESM2_ESM.pdf]

nature portfolio

Corresponding author(s): Haibin Shi

Last updated by author(s): Feb 18, 2022

Reporting Summary

Nature Portfolio wishes to improve the reproducibility of the work that we publish. This form provides structure for consistency and transparency in reporting. For further information on Nature Portfolio policies, see our Editorial Policies and the Editorial Policy Checklist.

Statistics

For all statistical analyses, confirm that the following items are present in the figure legend, table legend, main text, or Methods section.

n/a	Confirmed	
☐	☒	The exact sample size (n) for each experimental group/condition, given as a discrete number and unit of measurement
☐	☒	A statement on whether measurements were taken from distinct samples or whether the same sample was measured repeatedly
☐	☒	The statistical test(s) used AND whether they are one- or two-sided<br>*Only common tests should be described solely by name; describe more complex techniques in the Methods section.*
☐	☒	A description of all covariates tested
☐	☒	A description of any assumptions or corrections, such as tests of normality and adjustment for multiple comparisons
☐	☒	A full description of the statistical parameters including central tendency (e.g. means) or other basic estimates (e.g. regression coefficient) AND variation (e.g. standard deviation) or associated estimates of uncertainty (e.g. confidence intervals)
☐	☒	For null hypothesis testing, the test statistic (e.g. F, t, r) with confidence intervals, effect sizes, degrees of freedom and P value noted<br>*Give P values as exact values whenever suitable.*
☒	☐	For Bayesian analysis, information on the choice of priors and Markov chain Monte Carlo settings
☒	☐	For hierarchical and complex designs, identification of the appropriate level for tests and full reporting of outcomes
☐	☒	Estimates of effect sizes (e.g. Cohen's d, Pearson's r), indicating how they were calculated

Our web collection on statistics for biologists contains articles on many of the points above.

Software and code

Policy information about availability of computer code

Data collection	Not software was used for date collection.
Data analysis	All software used for date analysis are fully described in the materials and methods of manuscript. Fluorescence images of F720 were analyzed using the IVIS spectrum imaging system (PerkinElmer) and F1070 were analyzed using the NIR-II imaging system (Suzhou Yingrui Optical Technology Co., LTD., China). PA imaging was analyzed with a Multispectral Optoacoustic Tomography scanner (MSOT, iThera medical, Germany). Fluorescence images of cells and tumor slices were analyzed by ImageJ (1.53e). Statistical calculations were performed using OriginPro 2021 (64-bit).

For manuscripts utilizing custom algorithms or software that are central to the research but not yet described in published literature, software must be made available to editors and reviewers. We strongly encourage code deposition in a community repository (e.g. GitHub). See the Nature Portfolio guidelines for submitting code & software for further information.

Data

Policy information about availability of data

All manuscripts must include a data availability statement. This statement should provide the following information, where applicable:

- Accession codes, unique identifiers, or web links for publicly available datasets
- A description of any restrictions on data availability
- For clinical datasets or third party data, please ensure that the statement adheres to our policy

The experimental date supporting the findings of this study are available within the article and Supplementary Information. The date for all graphs generated in this study are provided in the source Date file. A reporting summary for this article is available as an Additional Information file.

Field-specific reporting

Please select the one below that is the best fit for your research. If you are not sure, read the appropriate sections before making your selection.

☒ Life sciences ☐ Behavioural & social sciences ☐ Ecological, evolutionary & environmental sciences

For a reference copy of the document with all sections, see nature.com/documents/nr-reporting-summary-flat.pdf

Life sciences study design

All studies must disclose on these points even when the disclosure is negative.

Sample size	No calculations were done to determine sample size. Sample sizes were determined according to precedent in previous literatures to enable statistical analyses such as standard deviation and t-tests. In vitro studies were repeated at least three times independently and in vivo sample sizes were determined based on standards for animal studies, attempting to have a minimum of N = 3 biological replicates with sufficient reproducibility.
Data exclusions	No data was excluded from this study.
Replication	Experiments were repeated at least three independent experiments with similar results. All experiments were reproduced to reliably support conclusions stated in the manuscript.
Randomization	For in vivo study, mice were inoculated with the same bath of tumor cells and randomly divided into experimental groups. No randomization was required for in vitro studies.
Blinding	Data acquisition and analyses were not blinded but all assays were performed at the same time for all groups of a given experiment. Since all conditions were subjected to the same analyses, blinding was not considered to be necessary.

Reporting for specific materials, systems and methods

We require information from authors about some types of materials, experimental systems and methods used in many studies. Here, indicate whether each material, system or method listed is relevant to your study. If you are not sure if a list item applies to your research, read the appropriate section before selecting a response.

Materials & experimental systems

n/a	Involved in the study	
☐	☒	Antibodies
☐	☒	Eukaryotic cell lines
☒	☐	Palaeontology and archaeology
☐	☒	Animals and other organisms
☒	☐	Human research participants
☒	☐	Clinical data
☒	☐	Dual use research of concern

Methods

n/a	Involved in the study	
☒	☐	ChIP-seq
☒	☐	Flow cytometry
☒	☐	MRI-based neuroimaging

Antibodies

Antibodies used	Anti -VEGF Rabbit (GB13034), Anti -CD31 Rabbit (GB113151) and Anti -KI67 mouse (GB13030-M-2) in Immunofluorescence staining was produced in Wuhan Xavier Biotechnology Co., LTD (China).
Validation	Alidation details of the primary antibodies are available on the manufacturers' websites: https://m.servicebio.cn/

Eukaryotic cell lines

Policy information about cell lines

Cell line source(s)	HCT116 human colon cancer cell and 293 [HEK-293] human embryonic kidney cells were purchased from from Stem Cell Bank, Chinese Academy of Sciences (Shanghai, China).
Authentication	All cell lines were authenticated by the supplier using Short Tandem Repeat test.
Mycoplasma contamination	All cell lines tested were negative for mycoplasma contamination.

Commonly misidentified lines (See ICLAC register)	All cell lines were not listed in commonly misidentified lines in ICLAC Register.

Animals and other organisms

Policy information about studies involving animals; ARRIVE guidelines recommended for reporting animal research

Laboratory animals	Balbc-Nu male mice were (~4-6 weeks old) were purchased from Changzhou Cavens Laboratory Animal Co., Ltd. (Jiangsu, China) and used for all the in vivo studies.
Wild animals	The study did not involve wild animals.
Field-collected samples	No field collected samples were used in the study.
Ethics oversight	Committee of Animal Research and Ethics in Soochow University.

Note that full information on the approval of the study protocol must also be provided in the manuscript.
